# Craniocervical junction involvement in inflammatory arthritis: a single-center radiologic study

**DOI:** 10.55730/1300-0144.5740

**Published:** 2023-11-11

**Authors:** Fatma YALÇINKAYA, Şafak PARLAK SAĞOL, Aynur AZİZOVA, Emre BİLGİN, Kader KARLI OĞUZ, Umut KALYONCU

**Affiliations:** 1 Department of Internal Medicine, Hacettepe University Faculty of Medicine, Ankara, Turkiye; 2 Department of Radiology, Hacettepe University Faculty of Medicine, Ankara, Turkiye; 3 Division of Rheumatology, Department of Internal Medicine, Hacettepe University Faculty of Medicine, Ankara, Turkiye

**Keywords:** Spondyloarthritis, rheumatoid arthritis, radiography, craniocervical junction

## Abstract

**Background/aim:**

Craniocervical junction (CCJ) can be involved in inflammatory arthritis. We aimed to define types of CCJ involvement in rheumatoid arthritis (RA), spondyloarthritis (SpA), and psoriatic arthritis (PsA) and compare them with patients without inflammatory arthritides.

**Materials and methods:**

In this retrospective analysis, cervical CT or MRIs of patients with RA, SpA, or PsA, taken for any reason between 2010 and 2020, according to ICD-10 codes, were scanned. Demographic data of the patients were recorded. CCJ involvements (atlantoaxial, vertical, or subaxial subluxation, odontoid process involvement) were reevaluated by an experienced radiologist. The control group consisted of consecutive patients without inflammatory arthritis.

**Results:**

Exactly 459 patients (204 RA, 200 SpA, and 55 PsA) and 78 patients in the control group were included in the study. CCJ involvement was detected in 101 (49.5%) RA, 53 (26.5%) SpA, 10 (18.2%) PsA, and 4 patients (5.1%) in the control group (p < 0.001). The odontoid process was one of the main targets, especially in RA patients (69 (33.8%)), which was significantly higher than in the SpA, PsA, and control groups. Although vertical subluxation (VS) was numerically higher in the RA and SpA groups compared to the control group, VS-related brainstem compression was relatively uncommon: 6 (2.9%) in RA, 1 (0.5%) in AS, and none in the PsA and control groups.

**Conclusion:**

CCJ involvement can often be detected in patients with inflammatory arthritis, especially in RA and SpA patients. The odontoid process is the main target of inflammation.

## 1. Introduction

Among inflammatory arthritis, rheumatoid arthritis (RA), spondyloarthritis (SpA), and psoriatic arthritis (PsA) can cause cervical spine involvement. Cervical spine involvement is given at the following frequencies in different studies: 45% in RA, 48%–54% in AS, and 35%–75% in PsA [1–4]. However, the craniocervical junction (CCJ) is a separate and unique area consisting of the atlantoaxial and atlantooccipital synovial joints. The 1st and 2nd cervical vertebrae have different features than other vertebrae. The C1 vertebra (atlas) consists of bilateral anterior and posterior arches connected by lateral masses. While the occipital condyles are located on the superior fovea articularis, located on the bilateral lateral masses, the inferior fovea articularis articulates with the C2 vertebra (axis). There are no discs between the occiput, atlas, and axis. The odontoid process is the other point of articulation of the axis with the atlas. It articulates with the anterior arch of the atlas, and the transverse ligament stabilizes this joint.

One of the most frequently affected areas in the cervical vertebrae in inflammatory arthritis is the CCJ [5]. Involvement can mainly be seen in 3 ways. The first and most common form is atlantoaxial subluxation (AAS), followed by vertical subluxation (VS) and subaxial subluxation (SAS). Although AAS can be detected in conventional cervical radiographs, evaluation of the CCJ, mainly by magnetic resonance imaging (MRI) or computed tomography (CT), provides more valuable results. Even though CCJ involvement in inflammatory arthritis has been shown separately in different studies, no study has compared the similarities and differences of this involvement type in RA, AS, and PsA. This study aimed to determine the frequency, distribution, differences, and related factors of craniocervical involvement in inflammatory arthritis detected by MRI or CT.

## 2. Materials and methods

### 2.1. Study population and patient selection

Patients diagnosed with RA, PsA, or SpA who underwent cervical vertebra CT and MRI for any reason at Hacettepe University’s Department of Internal Medicine, Division of Rheumatology, between June 2010 and October 2020 were included in this study. Between 2010 and 2020, 4442 patients with registered cervical CT or MR images and ICD-10 diagnosis codes of M05, M06, M07, or M45 were included in the initial examination. After duplications were excluded, 1558 patients remained. These patients’ medical records and medicine reimbursement reports were examined, and the diagnosis was confirmed. Finally, the diagnosis of 204 RA, 200 SpA, and 55 PsA patients were confirmed, and these patients were included in the study. To compare the frequency of CCJ involvement, the cervical MRIs of 240 consecutive patients who underwent MRI for any reason at Hacettepe University Hospital between April and June 2021 were examined. Following the exclusion of duplications, patients under 18 years of age, and patients diagnosed with inflammatory arthritis, 78 patients without inflammatory arthritis formed the control group.

### 2.2. Demographic, clinical, and laboratory characteristics of patients

The demographic characteristics of all patients were recorded. Accordingly, patients’ sex, age, age at imaging, and disease duration were noted. The diagnoses of the treating clinician were accepted, and no further examination was made.

Comorbid conditions and the smoking status of the patients were recorded. Accordingly, diabetes mellitus, hypertension, hyperlipidemia, chronic kidney disease, chronic liver disease, and malignancy were noted. The patients did not receive any treatment until imaging was recorded. Treatments were recorded as glucocorticoids, synthetic DMARDs (methotrexate, sulfasalazine, hydroxychloroquine, leflunomide), and biological DMARDs (anti-TNF, anti-IL6, B cell blockers, T cell blockers), and targeted synthetic DMARDs (tofacitinib). Acute phase responses, C-reactive protein, and erythrocyte sedimentation rates closest to the time of cervical imaging were recorded for all patients. Rheumatoid factor and the anti-cyclic citrullinated peptide (anti-CCP) antibody values of the RA patients were also noted.

### 2.3. Evaluation of cervical imaging

**Figure 1:**
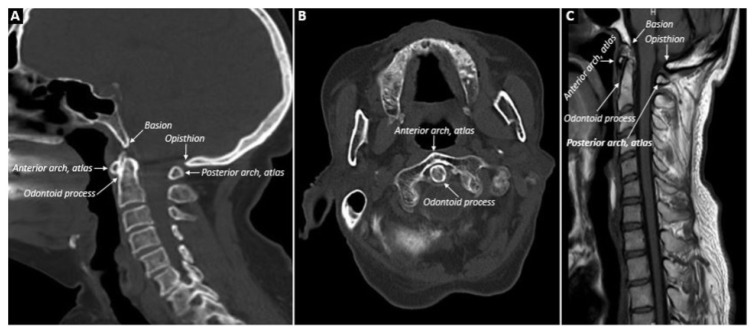
Normal anatomy of the cervical region is shown in **A, B** and **C**, **A**: sagittal CT; **B**: axial CT; **C**: sagittal MRI.

Conventional radiography, MRI, or CT were used to image the patients’ cervical region (Figure 1). The images were reevaluated by a radiologist (SP). If there was any doubt, the images were reevaluated by an experienced radiologist (KKO). The radiologists were unaware of the patients’ diagnoses during the evaluation. The most recent imaging was included in the study in patients with more than one cervical MRI and CT. If patients had both CT and MRI scans, both were evaluated. Reasons for performing cervical MRI or CT were noted: suspicion of disease involvement, head/neck pain, discopathy/radiculopathy, trauma, and other causes.

**Figure 2:**
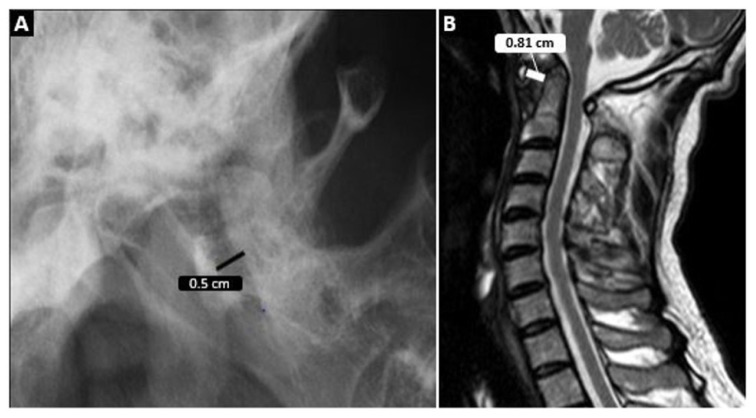
Atlantoaxial distance measurement on conventional radiography is seen in **A** and MRI in **B**.

In the radiological examination, the CCJ was evaluated. Cervical vertebral involvement was defined as the presence of at least one of the following radiological involvements: AAS, VS, SAS, odontoid process pathologies (resorption/pannus), atlantoaxial and atlantooccipital joint pathologies (narrowing, enlargement of the joint space, the presence of fluid, or synovitis in the joint space), and other abnormal findings (vertebral artery compression, C2 nerve root compression). AAS was divided into 4 types: anterior, posterior, lateral, and rotational. An AA space above 3 mm was considered anterior AAS (Figure 2). A diagnosis of posterior AAS was made when the posterior aspect of the anterior arch of C1 lay behind the anterior part of the C2 vertebral body. Lateral AAS was the unilateral involvement of the atlantoaxial joint where the lateral mass of C1 is displaced more than 2 mm laterally relative to C2 on coronal views. Rotational AAS was defined as the development of dislocation in the atlantoaxial joint due to unilateral destruction of the transverse ligament.

**Figure 3:**
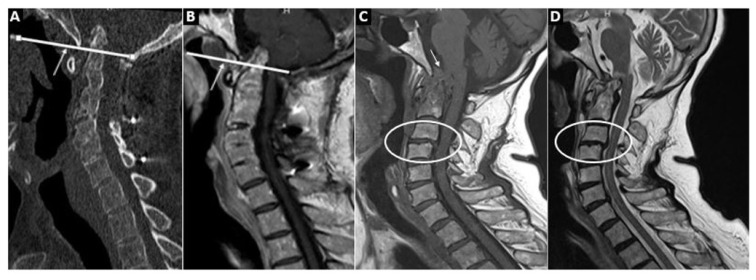
**A** (cervical CT) and **B** (cervical MRI) show the Chamberlain line and the associated vertical subluxation. In **C** (T1WI) and **D** (T2WI), basilar impression with pannus tissue at the craniovertebral junction causing stenosis of the foramen magnum and compression of the brainstem are shown. In addition, subaxial subluxation between C3–4 is seen.

For VS, the diagnosis was made when the length of the odontoid process over the Chamberlain line was more than 3 mm (Figure 3). The Chamberlain line is the line connecting the hard palate and the opisthion. If VS was detected, the length of the odontoid process above the Chamberlain line and brain stem compression, if present, were recorded. SAS is the slippage of one vertebra 2 mm or more from the adjacent vertebra at the levels below C2 (Figure 3).

The presence of erosion or pannus in the odontoid process in MRI and CT was noted (Figure 3). The radiologist visually evaluated atlantoaxial (C1 and C2 apophyseal) and atlantooccipital joint spaces, i.e. narrowing and widening (ŞP). The MRI specified suggested findings for synovial thickening and fluid in the atlantoaxial joint.

### 2.4. Statistical method

The SPSS (Version 25 for MacOS) program was used for statistical analysis. The conformity of the variables to normal distribution was examined using visual (histogram and probability graphs) and analytical methods (Kolmogorov–Smirnov). Numerical (continuous or discrete) variables did not have a normal distribution. Therefore, these variables were presented using the descriptive statistics of median and minimum–maximum values; intergroup comparisons were made using the Mann–Whitney U test. Where necessary, whether there was a difference in frequency between the patient and control groups in the categorical data was compared using chi-square and Fisher tests. In multivariable analysis, independent predictors of differential diagnosis between the 2 groups using possible factors (variables with p < 0.20) identified in previous analyses were examined using logistic regression analysis. The Hosmer–Lemeshow test was used for goodness of fit for logistic regression models. A type-1 error level below 5% was considered statistically significant. The study was registered with GO 20/1102 and approved by Hacettepe University’s Non-Interventional Clinical Research Ethics Committee on 17.11.2020 (decision no. 2020/19-41).

## 3. Results

### 3.1. General information

Four hundred fifty-nine patients with inflammatory arthritis (73% female) were included in the study. Of these patients, 204 (44.4%) had RA, 200 (43.6%) had SpA, and 55 (12.0%) had PsA. The median age at diagnosis was 44 (14–88) years. The median time to diagnosis at the time of imaging was 4 years. Imaging was performed at the earliest 7 years before and at the latest 39 years after diagnosis. The most common reason for imaging was head and neck pain, which was present in 141 patients (30.7%) (Table 1).

The number of patients who underwent cervical imaging before diagnosis was 40/459 (8.8%). The distribution of patients who underwent cervical imaging before diagnosis is as follows: 19/40 (47.5%) RA, 12/40 (30.0%) SpA, and 9/40 (22.5%) PsA. In the imaging studies, VS was detected in 4/40 (10.0%) (1 RA, 1 SpA, 2 PsA), SAS in 2 (5%) (1 RA and 1 SpA), resorption of the odontoid process in 4/40 (10%), and pannus (soft tissue surrounding the odontoid process) in 1/40 (2.5%) patients.

### 3.2. Craniocervical junction involvement in inflammatory arthritis

CCJ involvement was seen in 101 (49.5%) RA patients, 53 (26.5%) SpA patients, and 10 (18.2%) PsA patients (p < 0.001). Only 5.1% of the patients in the control group had CCJ involvement. CCJ involvement was more common across all inflammatory arthritis groups than in the control group (p < 0.001) (Tables 2 and 3). The distribution of CCJ involvement according to inflammatory arthritis subtypes is shown in Tables 2 and 3.

The median length of the odontoid process above the Chamberlain line was 5.25 mm (3.40–12.3) in RA, 4.6 mm (3.4–12.0) in SpA, and 4.3 mm in PsA (4.0–7.8); there was no significant difference between the groups (p = 0.43). AAS was less common in the RA and AS groups (7.8% and 5.0%, respectively) than in patients with VS. AAS was detected in only 2/55 (3.6%) of the PsA patients. SAS had a relatively rare involvement rate (between 1.8% and 5.9%). Among RA patients, spinal cord compression was observed in 5 (29.4%) patients with AAS. Brain stem compression was detected in 6/24 (25.0%) patients with VS. Of the patients with SpA, only one (1.9%) of 53 patients with CCJ involvement had spinal cord compression. Both AAS and SAS were detected in the patient with spinal cord compression. None of the PsA patients had spinal cord compression.

### 3.3. Craniocervical junction involvement in rheumatoid arthritis

CCJ involvement was detected in 101 (49.5%) of 204 RA patients included in the study. Patients with CCJ involvement were older at the time of diagnosis (53 (19–88) years versus 48 (18–85) years; p = 0.034). In comparison, there was no difference between male and female RA patients regarding the presence of AAS, VS, and SAS. Odontoid process involvement ((21/38 (55.3%) vs. 48/166 (28.9%); p = 0.002), pannus in the odontoid process ((13/38 (34.2%) vs. 24/166 (14.5%); p = 0.004), and odontoid process resorption ((18/38 (47.4%) vs. 44/166 (26.0%), 5); p = 0.012) were detected more frequently in male RA patients. It was noticed that in RA patients with radiological involvement, the erythrocyte sedimentation rate (ESR) ((24 (2–120) vs. 14 (3–86); p < 0.001) and CRP ((0.9 (0.1–26.4) vs. 0.5 (0.1–16); p = 0.001) values were significantly higher than in RA patients without radiological involvement. In the multivariable analysis performed to investigate the factors associated with CCJ in patients with RA, age at diagnosis (for each unit increase) (OR (95% confidence interval (CI)): 1.03 (1.01–1.06); p = 0.008), disease duration (per unit increment) (1.06 (1.02–1.10)); p = 0.004), C-reactive protein (per unit increase) (1.11 (1.02–1.22); p = 0.023) were found to be the independent predictors.

According to cervical MRI/CT evaluation, AAS was detected in 17 (8.3%) RA patients. Thirteen of the patients with AAS were female (76.5%). While age at diagnosis was similar in patients with and without AAS, disease duration was longer in patients with AAS ((12 (1–25) vs. 5 (–5 to 39); p = 0.022). Disease duration (for each unit increase) (OR (95% CI): 1.05 (1.01–1.11); p = 0.043), and ESR (for each unit increase) (1.02 (1.00–1.04); p = 0.050) were independently associated with the presence of AAS in RA patients in multivariate analysis. While the atlantoaxial distance was 1.4 (0–8.0) mm in all RA patients, it was 3.2 (1.0–8.0) mm in patients with AAS.

Odontoid process pathologies, especially odontoid resorption, were found to be more common in anti-CCP positive patients when compared to that of anti-CCP negative patients (35/81 (43.2%) vs. 14/61 (23.0%); p = 0.012). In the multivariate analysis investigating the factors associated with the involvement of the odontoid process in RA patients, the male sex (OR (95% CI)): 2.93 (1.14–7.54); p = 0.025), CRP (per unit increment) (1.12 (1.01–1.24)); p = 0.031), and anti-CCP positivity (2.75 (1.26–5.98); p = 0.011) were found to be independently associated. Rheumatoid factor positivity did not affect CCJ involvement. AAS was more common in patients with pannus in the odontoid process (24.3% vs. 4.2%, p < 0.001).

### 3.4. Craniocervical junction involvement in spondyloarthritis and psoriatic arthritis

CCJ involvement was detected in 53 of 200 patients (26.5%) with SpA diagnosis. Of the patients with involvement, 25/53 (47.2%) were male. The median age at diagnosis of SpA patients with involvement was 39 (19–73) years, and there was no difference between those with and without involvement. CCJ involvement was detected in 10 (18.2%) of the 55 PsA patients included in the study. Eight (80%) of the patients with involvement were female. The median age at diagnosis of the patients with involvement was 45 (33–66) years, and there was no difference between those with and without involvement.

Odontoid process pathologies were more common in male SpA patients than in females (14/76 (18.4%) vs. 19/124 (8.1%); p = 0.029). The use of glucocorticoids was higher in patients with AAS than in patients without it (44.4% vs. 11.2%, p = 0.004); there was no difference between the groups in other respects. Steroid use was more common in PsA patients with CCJ involvement than in those without (70% vs. 33.3%; p = 0.03).

## 4. Discussion

The CCJ is an “overlooked” joint area difficult to assess anatomically, and patients’ symptoms were unclear. The cervical region is even more neglected, especially in a disease like RA, where peripheral joints are prioritized. On the other hand, in SpA patients with cervical region involvement, entheseal/bony changes in the cervical vertebrae come to the forefront. Consistent with the literature, CCJ pathologies were primarily found in RA patients and less frequently in SpA and PsA patients.

One of the most important findings of our study was that the odontoid process was involved in one-third of RA patients. Resorption in the odontoid process was also detected in almost all patients whose odontoid bone was affected. In addition, the pannus tissue was seen in the odontoid process in approximately one-fifth of all RA patients. Possible risk factors associated with the involvement of the odontoid process in RA patients were also investigated in our study. In multivariable analysis, it was found that the male sex (approximately 3 times), CRP elevation (1.1 times), and anti-CCP positivity (about 2.5 times) increased the risk of odontoid process involvement. In their study, Olah et al. evaluated 49 female RA patients in terms of AAS and odontoid process erosion and compared 8 patients with odontoid process erosion and 41 patients without erosion—and found no difference in terms of CRP, anti-CCP positivity, and ESR values [6]. It is well known that anti-CCP positivity is associated with erosive disease in RA. In our study, when the relationship between anti-CCP and the odontoid process was evaluated in this respect, it provided us with important guiding information. According to our results, the following inference can be made: One of the possible anatomical target areas in RA patients is the odontoid process and, in anti-CCP positive male patients, especially if the acute phase response is high, involvement of the odontoid process should always be considered by the clinician.

AS is the first disorder that comes to mind when CCJ involvement is mentioned in RA patients. In their meta-analysis published in 2015, Zang and Pope stated that AAS was the most common form of involvement [1]. Accordingly, 27% of 2737 RA patients had AAS, mainly anterior AAS. These results contradict the approximately 8% AAS rate in our RA patients. However, when we look at the data, several reasons exist for these contradictory results. Namely, our study’s mean disease duration in RA patients was 5 years. In addition, our multivariable analysis determined that the most critical factor determining the development of AAS in RA was the duration of the disease. The disease duration of patients with AAS was also considerably longer than those without AAS (approximately 11.5 vs. 4.5 years). Although the frequency of AAS was 27% in the meta-analysis, the mean disease duration of these patients was 12 years. In the same meta-analysis, the rate of progression of AAS was also calculated, and it was found that 4 out of 100 patients showed a progression of AAS every year. Based on these results, our study’s relatively low AAS rates may be associated with short-term follow-up.

It is alarming that the relationship between the odontoid process and the C1 arch is disrupted and that the odontoid process is dislocated vertically toward the brain stem. Fatal medullary compression was seen in 10% of RA patients in a postmortem study [7]. These rates suggest that VS is not uncommon in RA patients. Our research also detected VS in approximately 12% of RA patients. In the meta-analysis results of CCJ involvement in RA mentioned above, VS was found in 11% of the patients (95% CI; 10%–19%) [1]. This rate is quantitatively identical to the results of our study.

Interestingly, our study showed VS at high rates in RA, SpA, and PsA patients. More information about VS in SpA and PsA is needed. Some crucial differences between the VSs occur in RA and SpA/PsA. One of the most important differences is the spinal cord/medulla compression. While a quarter of patients with VS due to RA have spinal cord compression, this rate was relatively low in patients with SpA and absent in PsA. This may be related to the different VS development mechanisms in RA and SpA. Prospective controlled studies should investigate the underlying mechanisms of VS in RA and SpA patients.

Another interesting result was found in our study. Cervical MRI/CT was performed before diagnosis in 40 of 459 inflammatory arthritis patients included in the study. Therefore, cervical imaging was performed, possibly in the “pre-clinical period” in these patients. We can make this process more efficient, especially in RA patients. In a small case series published in 2014, the information of 3 patients who may have had CCJ involvement before the development of the RA clinic was presented [8].

Unfortunately, studies on CCJ involvement in patients with SpA are limited compared to RA. In a study by Ramos-Remus et al. published in 1995, AAS was found in 21% and VS in 2% of the conventional cervical radiographs of 103 AS patients [9]. The CCJ was not evaluated with advanced imaging methods in that study. Conventional cervical radiographs of our patients were also assessed, and very low rates were found compared to the study mentioned above. In a 2001 study published by Lee et al., 112 AS patients with a disease duration of approximately 11 years were evaluated [2]. In that study, the frequency of AAS was 11.7%, so it can be considered methodologically more consistent. In the present study, the frequency of AAS was 5% in SpA patients with a disease duration of 4 years. There are few detailed studies on odontoid process involvement, VS, atlantoaxial, and atlantooccipital joint involvement in SpA patients. Our study is valuable because it shows different components of craniocervical involvement in SpA. Remarkably, the odontoid process may be involved, although not as much as RA. Studies on CCJ involvement in PsA are much less common. In a study from 1992, AAS involvement was found in 23% of 57 PsA patients in the evaluation based on conventional radiographs [4]. However, these rates are quite misleading; the study was performed on a select group of patients. In a study by Queiro et al. from 2002, AAS was detected in only one of 100 PsA patients evaluated with conventional radiography [10]. In the literature, it is striking that further studies in which evaluation was performed using MRI/CT are not available in PsA patients. In our study, the frequency of AAS was found to be only 3.6%, according to MRI/CT.

The most important limitation of our study was its retrospective nature. It was impossible to further comment on the patients’ activity during the imaging period, drug use, functional status, and neurological symptoms. Appropriate neurological examinations and inquiries could have provided valuable information, especially during advanced imaging. Therefore, there is a clear need for a long-term prospective study, especially in RA patients. Another shortcoming is that the abnormalities in the CCJ were not compared with a control group of similar age and sex. The strength of our research was the evaluation of the CCJ with cross-sectional imaging methods in a very large patient group. Reevaluation of all images by the radiologists who were blinded to the clinical findings of the patients strengthens our study. Indeed, it is impossible to establish valid comparisons across the 3 studied diseases when patients differ in age and in terms of disease duration. However, a comparison of RA, SpA, and PsA, which may affect the CCJ by a similar methodology, has never previously been attempted and significantly adds to the literature.

In conclusion, CCJ involvement was detected in 101 (49.5%) RA, 53 (26.5%) SpA, 10 (18.2%) PsA, and 4 patients (5.1%) in the control group. The odontoid process was one of the main affected regions, affecting 33.8% of RA patients. In SpA patients, enteseal/bony changes in the cervical region were more commonly encountered. The cervical region is a difficult-to-assess area and should be evaluated more vigorously by clinicians.

## Figures and Tables

**Table 1:** Characteristics of rheumatoid arthritis, spondyloarthritis, and psoriatic arthritis patients.

	All groups (n = 459)	Rheumatoid arthritis (n = 204)	Spondyloarthritis (n = 200)	Psoriatic arthritis (n = 55)	p: RA vs. SpA vs. PsA
**Female, n (%)**	335 (73.0)	166 (81.4)	124 (62.0)	45 (81.8)	**<0.001**
**Age at diagnosis** *	44 (14–88)	50 (41–60)	38 (32–47)	45 (35–52)	**<0.001**
**Duration of disease at the time of CT/MRI** *	4 (−7 to 39)	5 (−5 to 39)	4 (−4 to 35)	2 (−7 to 35)	**0.046**
**Reason for imaging, n (%)**					0.10
Suspicion of disease involvement	90 (19.6)	30 (14.7)	43 (21.5)	17 (30.9)	
Head and neck pain	141 (30.7)	61 (29.9)	70 (35)	10 (18.2)	
Discopathy–radiculopathy	131 (28.5)	68 (33.3)	47 (23.5)	16 (29.1)	
Trauma	41 (8.9)	17 (8.3)	19 (9.5)	5 (9.1)	
Other/unknown	56 (12.2)	28 (13.7)	21 (10.5)	7 (12.7)	
**Comorbidity, n (%)**					0.52
DM	80/458 (17.5)	46/203 (22.7)	20/199 (10.1)	14 (25.5)	
HT	159/458 (34.7)	93 (45.6)	46/199 (23.1)	20 (36.4)	
HL	74/458 (16.2)	35 (17.2)	31/199 (15.7)	8 (14.5)	
CKD	13/458 (2.8)	9 (4.4)	3/199 (1.5)	1 (1.8)	
Malignancy	20/458 (4.4)	14 (6.9)	5/199 (2.5)	1 (1.8)	
Other	253/458 (55.4)	91 (44.6)	133/199 (67.2)	29 (52.7)	
**Smoking, n (%)**					**0.007**
Never smoked	126 (27.5)	62 (30.4)	54 (27.0)	10 (18.2)	
Active or former smoker	158 (34.4)	53 (26)	78 (39.0)	27 (49.1)	
Unknown	175 (38.1)	89 (43.6)	68 (34)	18 (32.7)	
**Drugs, n (%)**					
Glucocorticoid	266/454 (58.6)	142/203 (69.6)	25/196 (12.8)	22 (40)	**<0.001**
csDMARD	371/454 (81.7)	179/203 (88.2)	150/196 (76.5)	42 (76.4)	**0.006**
Methotrexate	200/452 (44.2)	114/203 (56.2)	50/194 (25.8)	36 (65.5)	**<0.001**
Leflunomide	99/452 (21.9)	82/202 (40.6)	7/195 (3.6)	10 (18.2)	**<0.001**
Sulfasalazine	246/453 (54.3)	81/203 (39.9)	139/195 (71.3)	26 (47.3)	**<0.001**
Hydroxychloroquine	195/454 (43)	140/203 (69)	42/196 (21.4)	13 (23.6)	**<0.001**
bDMARD	135/454 (29.7)	39/203 (19.2)	72/196 (36.7)	24 (43.6)	**<0.001**
tsDMARD	3/203 (1.5)	3/203 (1.5)	N/A	N/A	N/A

DM: type-2 diabetes mellitus; HT: hypertension; HL: hyperlipidemia; CKD: chronic kidney disease; csDMARD: conventional DMARD; bDMARD: biological DMARD; tsDMARD: targeted synthetic DMARD.

* Data show median value (lowest–highest).

**Table 2:** Comparison of types of craniocervical involvement in the subgroups and control group.

n (%)	RA (n = 204)	SpA (n = 200)	PsA (n = 55)	Healthy control (n = 78)	p1: RA vs. SpA vs. PSA	p2: RA vs. SpA	p3: RA vs. PsA	p4: SpA vs. PsA
Any type of involvement	101 (49.5)	53 (26.5)	10 (18.2)	**4 (5.1)**	**<0.001**	**<0.001**	**<0.001**	0.20
Odontoid process involvement	69 (33.8)	24 (12)	2 (3.6)	**0**	**<0.001**	**<0.001**	**<0.001**	0.07
Odontoid process resorption	62 (30.4)	19 (9.5)	2 (3.6)	**0**	**<0.001**	**<0.001**	**<0.001**	0.27
Odontoid process pannus	37 (18.1)	9 (4.5)	2 (3.6)	**0**	**<0.001**	**<0.001**	**0.008**	0.78
Atlantoaxial subluxation	17 (8.3)	10 (5)	2 (3.6)	0	0.27	0.18	0.38	0.98
Spinal cord compression due to AAS	5 (2.5)	1 (0.5)	0	0	0.15	0.21	0.59	1.0
Vertical subluxation	24 (11.8)	20/197 (10.2)	6 (10.9)	3 (3.8)	0.88	0.61	0.86	0.87
Brainstem compression due to VS	6 (2.9)	1 (0.5)	0	0	0.08	0.12	0.35	1.0
Subaxial subluxation	12 (5.9)	4 (2)	1 (1.8)	1 (1.8)	0.09	0.045	0.22	0.93
Spinal cord compression due to SAS	3 (1.5)	1 (0.5)	0	0	0.44	0.62	0.99	1.0
Atlantoaxial joint involvement	23/186 (12.4)	13 (6.5)	2 (3.6)	0	0.045	0.048	0.06	0.50
Atlantooccipital joint involvement	6/187 (3.2)	3 (1.5)	0	0	0.26	0.32	0.34	0.84
Other	9 (4.4)	3 (1.5)	0	0	0.08	0.08	0.11	0.83

AAS: atlantoaxial subluxation; VS: vertical subluxation; SAS: subaxial subluxation; RA: rheumatoid arthritis; SpA: spondyloarthritis; PsA: psoriatic arthritis.

**Table 3:** Comparison of types of craniocervical involvement in the rheumatoid arthritis, spondyloarthritis, and control groups.

	RA (n = 204)	SpA (n = 200)	Healthy control (n = 78)	p: RA vs. SpA vs. control	p: RA vs. control	p: SpA vs. control	p: RA vs. SpA
**Female, n (%)**	166 (81.4)	124 (62)	55 (70.5)	<0.001	0.048	0.18	<0.001
Age at the time of CT	50 (41–60)	38 (32–47)	46 (18–86)	<0.001	<0.001	0.45	<0.001
Any type of involvement	101 (49.5)	53 (26.5)	4 (5.1)	<0.001	<0.001	<0.001	<0.001
Odontoid process involvement	69 (33.8)	24 (12)	0	<0.001	<0.001	0.001	<0.001
Odontoid process resorption	62 (30.4)	19 (9.5)	0	<0.001	<0.001	0.005	<0.001
Odontoid process pannus	37 (18.1)	9 (4.5)	0	<0.001	<0.001	0.065	<0.001
Atlantoaxial subluxation	17 (8.3)	10 (5)	0	0.022	0.004	0.067	0.018
Spinal cord compression due to AAS	5 (2.5)	1 (0.5)	0	0.12			
Vertical subluxation	24 (11.8)	20/197 (10.2)	3 (3.8)	0.13			
Brainstem compression due to VS	6 (2.9)	1 (0.5)	0	0.06			
Subaxial subluxation	12 (5.9)	4 (2)	1 (1.3)	0.054			
Spinal cord compression due to SAS	3 (1.5)	1 (0.5)	0	0.38			
Atlantoaxial joint involvement	23/186 (12.4)	13 (6.5)	0	0.002	0.001	0.023	0.32
Atlantooccipital joint involvement	6/187 (3.2)	3 (1.5)	0	0.19			

AAS: atlantoaxial subluxation; VS: vertical subluxation; SAS: subaxial subluxation; RA: rheumatoid arthritis; SpA: spondyloarthritis; PsA: psoriatic arthritis.
